# Airborne Aerosolized Mouse Cytomegalovirus From Common Otolaryngology Procedures: Implications for COVID-19 Infection

**DOI:** 10.1177/0194599820957966

**Published:** 2020-09-15

**Authors:** Tofigh Sayahi, Christopher Nielson, Yuan Yu, Kaden Neuberger, Michael Seipp, Matthew A. Firpo, Kerry Kelly, Albert H. Park

**Affiliations:** 1 Department of Chemical Engineering, University of Utah, Salt Lake City, Utah, USA; 2 Division of Otolaryngology–Head and Neck Surgery, University of Utah, Salt Lake City, Utah, USA; 3 Department of Surgery, University of Utah, Salt Lake City, Utah, USA

**Keywords:** cytomegalovirus, COVID-19, airborne, aerosolization, aerosol-generating procedure, otolaryngology surgical procedures

## Abstract

**Objectives:**

To determine whether common otolaryngology procedures generate viable aerosolized virus through a murine cytomegalovirus (mCMV) model for infection.

**Study Design:**

mCMV model of infection.

**Setting:**

University of Utah laboratory.

**Methods:**

Three-day-old BALB/c mice were inoculated with mCMV or saline. Five days later, each mouse underwent drilling, microdebrider, coblation, and electrocautery procedures. Particle size distribution and PM_2.5_ (particulate matter <2.5 µm) concentration were determined with a scanning mobility particle sizer and an aerosol particle sizer in the range of 15 nm to 32 µm. Aerosolized samples from these procedures were collected with an Aerosol Devices BioSpot sampler for viral titer based on polymerase chain reaction and for viable virus through viral culture.

**Results:**

As compared with the background aerosol concentrations, coblation and electrocautery showed statistically significant increases in airborne aerosols (Tukey-adjusted *P* value <.040), while microdebrider and drilling at 30,000 rpm did not (.870 < Tukey-adjusted *P* value < .930). We identified viral DNA in samples from coblation and drilling procedures, although we did not identify viable viruses in aerosol samples from any of the 4 procedures.

**Conclusion:**

Coblation and electrocautery procedures generate >100-fold increases in aerosol concentrations over background; only coblation and drilling produce aerosolized viral DNA. The high concentration of aerosols from coblation and electrocautery suggests the need for appropriate safeguards against particle exposure to health care workers. The presence of viral DNA from drilling and coblation procedures warrants the need for appropriate protection against droplet and aerosol exposure.

The COVID-19 pandemic started in Wuhan, China, last December 2019. According to the Johns Hopkins Coronavirus Resource Center, 17,639,185 people have been infected with this disease and 680,575 have died as of August 1, 2020.^1^ This infection has significantly affected society through quarantines, stay-at-home orders,^2^ business closures, and travel prohibitions. In April 2020, the US unemployment rate jumped to 14.7%, the highest level since the Great Depression.^3^

A key priority during this crisis has been the need to minimize disease transmission to health care personnel. As millions of people have been staying at home to minimize viral transmission, health care workers have been doing the opposite in going to hospitals and clinics. Several reports indicate that the SARS-CoV-2 virus particles reside with extremely high concentrations in the oral cavity and nasopharynx and can be a significant source of transmission.^4-6^ This characteristic property of the virus places health care professionals who examine and work in these areas at particular risk. Otolaryngologists and ancillary staff are especially vulnerable to viral transmission directly through mucus, blood, and aerosolized particles when examining or operating in these areas. There have been anecdotal reports from China, Italy, and Iran that otolaryngologists are among the highest-risk group contracting the virus while performing upper airway procedures and examinations if not using appropriate personal protective equipment.^7-9^ This dilemma puts otolaryngologists in a difficult situation when presented with patients with time-sensitive and emergent problems that require surgery.

Two studies evaluated several endonasal and otologic procedures. Using fluorescein solution and digital image processing in a cadaveric head model and clinical setting, the authors noted that only high-speed drilling produced significant aerosol contamination.^10,11^ We proposed to build on these studies by measuring particle size and concentration and by trying to detect aerosolized viral DNA and viable virus during common otolaryngology procedures, using a murine model for cytomegalovirus (CMV) infection.

## Materials and Methods

### Viruses

Recombinant murine CMV (mCMV; strain K181 MC.55 [ie2– GFP+]) expressing green fluorescent protein (GFP) was used. Virus purification was carried out by Virapur as previously described.^12^

### Animals

Inbred BALB/c mice were used for these experiments. Mice were housed and bred under specific pathogen–free conditions under controlled temperature and humidity at the Central Animal Facility at the University of Utah. The University of Utah Institutional Animal Care and Use Committee approved all procedures in accordance with the standards established by the US Animal Welfare Act.

### Viral Inoculation

Mice were injected via an intracerebral route with recombinant mCMV (strain K181 MC.55 [ie2– GFP+]) expressing GFP at postnatal day 3 as previously described.^12^ Control animals (uninfected) received the same volume of normal saline. The injections were completed with a 5-μL Hamilton syringe with a 30G needle.

### Surgical Procedures

Mice underwent the following procedures in order: drilling (at 30,000 rpm), microdebrider, coblation (setting at 7 for coblate), and electrocautery (with and without suction) at 8 days of age. Animals were anesthetized with xylazine/ketamine. The hair over the cranium was shaved. A 1-cm incision was made to expose the skull, which was removed with an otologic drill and saline irrigant (Acumed). The brain was then removed with a 3.4-mm Tricut Blade Microdebrider (Medtronics). The remaining soft tissue in the head and neck, chest, and abdomen was removed with a coblator (Smith & Nephew) and then with a monopolar bovie set (Ethicon Megadyne) at 15 W. Each procedure was performed for 6 minutes 45 seconds. An additional 4 minutes 30 seconds elapsed before the next procedure to ensure that aerosolization dropped to background levels. This step was verified by real-time measurements of aerosol concentration.

### Aerosol Measurements

Particle size distribution and concentration were measured with a scanning mobility particle sizer and an aerosol particle sizer (TSI SMPS 3081–TSI APS 3022; SMPS-APS) in the range of 15 nm to 20 µm. Each SMPS-APS scan required 2 minutes 15 seconds, and 3 scans were performed for each procedure (total, 6 minutes 45 seconds). In addition, a GRIMM 1.109 aerosol spectrometer provided PM_2.5_ (particulate matter <2.5 µm) mass concentration and size distribution in the range of 0.225 to 34 µm in 31 class sizes in 6 seconds. A TSI DustTrak II 8530 provided additional PM_2.5_ mass concentration measurements in 6 seconds. The measurements were collected in the breathing zone within 25 cm of the procedure. Background measurements were collected 15 minutes prior to each procedure, during each procedure (6 minutes 45 seconds), and 15 minutes postprocedure, which allowed us to determine the aerosol decay time.

### Virus Titers and Viral Load

We collected 1 liquid sample for each surgical procedure and each mouse for a total of 17 samples for each procedure with a Liquid Spot sampler (SLL 110A; Aerosol Devices). The liquid contained 2% bovine serum albumin in 1% sterile phosphate-buffered saline (Thermo Fisher), and these samples were analyzed for mCMV. The Spot employs a condensation growth tube and allows for >90% virus collection efficiency and improved virus viability as compared with traditional samplers, which have virus collection efficiencies of <10%.^13-15^

### Detection of mCMV Genome by qPCR

The presence of viral DNA was assessed in aerosol condensates and tissue culture by quantitative polymerase chain reaction (qPCR) with the CMV immediate-early response gene 1 (IE1) as the amplification target, as previously described.^16^ DNA was purified from samples with the Quick-DNA Miniprep Kit (Zymo Research). Each sample was assayed in duplicate with the Taqman Gene Expression Master Mix (Life Technologies), IE1 primers and IE1 Prime Time qPCR Probe (Integrated DNA Technologies), and the Applied Biosystems QuantStudio 12K Flex Real Time PCR System (Life Technologies). Quantification of IE1 was achieved by comparing the crossing threshold of the unknown samples to that of an IE1 standard curve. The IE1 standard curve was created by extracting DNA from a solution of stock mCMV in 2% bovine serum albumin/phosphate-buffered saline and quantified with Qubit fluorometric quantification (Thermo Fisher). A copy number log dilution series standard curve was generated to calculate amplification efficiency relative to the theoretical maximum. The observed efficiency was applied to the lowest reproducible dilution to assign the limit of detection.

### Assessment of Infectious CMV in Tissue Culture

To assess the presence of infectious CMV viral particles, cultured cells were inoculated with aerosol condensates and monitored for GFP expression by fluorescent microscopy. NIH-3T3 cells (ATCC) were seeded onto 96-well plates and grown overnight to 70% confluence. Ten microliters of each condensate sample and positive control standards were applied in duplicate and incubated for 2 hours at 37 °C and 5% CO_2_. The standard consisted of 4 log dilutions per well (63,000, 6300, 630, and 63 pfu). After the 2-hour incubation, 70 µL of fresh media was added, and the plates were incubated for 24 hours before microscopic examination. Imaging was performed on a Nikon A1 Confocal Microscope with a GFP laser and brightfield imaging (Cell Imaging Core, University of Utah).

The presence of infectious CMV viral particles was also monitored by qPCR. Twenty-four–well plates were seeded with 5 × 10^5^ NIH-3T3 cells and grown overnight to achieve 70% confluence. All but 100 µL of media was removed from the 24-well plates, and 10 µL of sample was applied in duplicate wells. After 2-hour incubation at 37 °C and 5% CO_2_, 900 µL of fresh media was added, and the plates were then incubated for an additional 72 hours. Prior to DNA extraction, the covered plates were examined with an EVOS microscope and GFP laser for fluorescence (Cell Imaging Core, University of Utah). In 1 experiment, culture supernatant was removed and viral DNA quantified in the adherent cells by qPCR. In a separate experiment, viral DNA was assessed in cells and supernatant to capture signal from any shed virus. In both cases, adherent cells were freed from the wells with 0.25% Trypsin (Gibco). DNA was extracted as described earlier.

### Statistical Analyses

The analyses were performed in Python 3. The measurements from each instrument were averaged over the period for each mouse and each procedure (6 minutes 45 seconds). Student’s *t* test was used to determine the statistical differences between the uninfected and infected tests. Tukey’s test was performed to evaluate the statistical differences between the blank test and each procedure.^17^ The aerosol sampling instrumentation measures particle size and concentration with accuracies of 5% and 2%, respectively.^18,19^ The Spot sampler has a standard deviation of 593 pfu/L of viable MS2 virus.^14^ We anticipated that the Spot would have similar standard deviation for CMV.

## Results

### Aerosol Measurements

**Figure 1.**
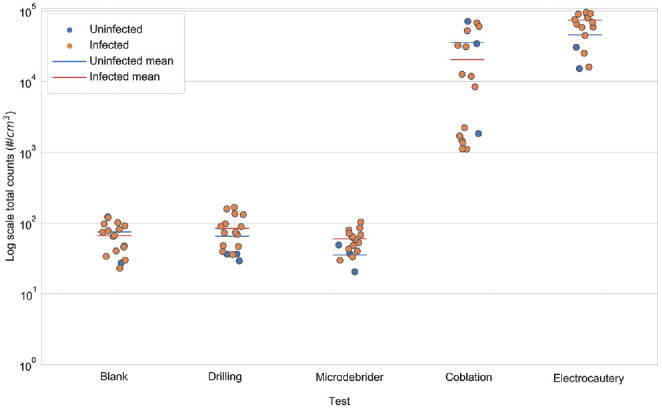
Comparison of SMPS-APS total particle counts between 3 uninfected and 14 infected samples. The bar denotes the mean total count. SMPS-APS, scanning mobility particle sizer.

**Figure 2.**
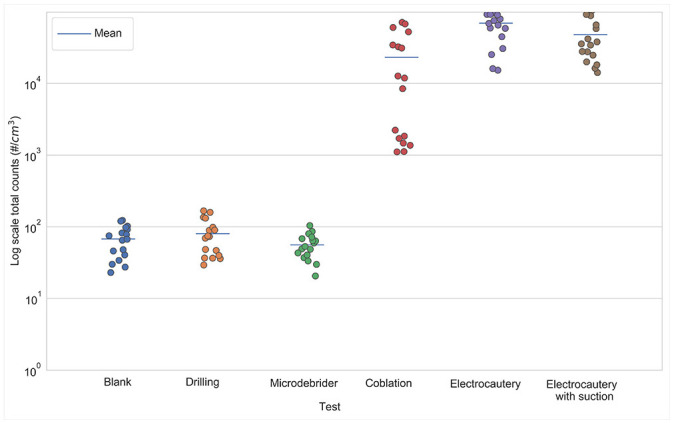
SMPS-APS total particle counts for each procedure. The bar denotes the mean total count. SMPS-APS, scanning mobility particle sizer and an aerosol particle sizer.

**Figure 3.**
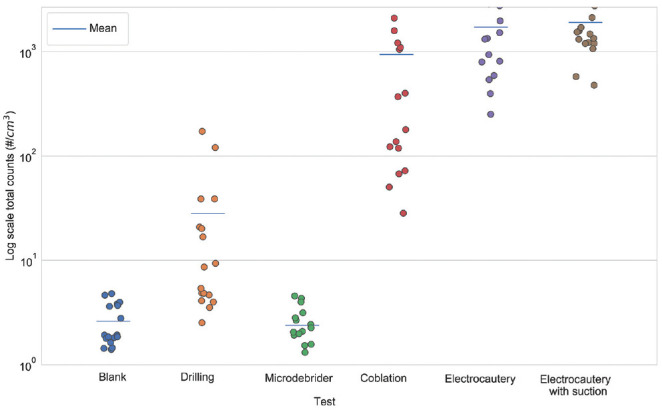
Grimm total particle counts for all procedures. The bar denotes the mean total count.

We examined the difference between aerosol generation in infected and uninfected mice. **Figure 1** and **Table 1** show that, in general, the total count and PM_2.5_ concentration differences between the uninfected and infected mice were not statistically significant (.067 < *P* values < .970, except for microdebrider Grimm total counts and drilling SMPS-APS total counts, which were close to the background counts). Therefore, we analyzed the aerosol measurements from all 17 mice for each procedure, regardless of infection status. Two procedures, coblation and electrocautery, generated the highest concentrations of aerosols (**Table 2**; Supplemental Figures S1 and S2, available online). For example, the SMPS-APS showed 364- and 1202-fold increases in total particle counts for coblation and electrocautery, respectively, as compared with background. During electrocautery, suction reduced the total count by 26.8%; however, this reduction was not statistically significant (*P* = .082). The results also indicated that drilling and microdebrider did not cause statistically significant increases in aerosol concentrations and total counts when compared with background (.870 < Tukey-adjusted *P* value < .930; **Figures 2** and **3**, **Table 3**).

**Table 1.** Student’s *T*-Test *P* Values Comparing the Measurements During Several Surgical Procedures From 3 Uninfected and 14 Infected Mice.

Test	DustTrak PM_2.5_	Grimm PM_2.5_	Grimm total counts	SMPS-APS total counts
Blank	.227	.970	.924	.787
Drilling	.444	.218	.258	<.001
Microdebrider	.631	.370	<.001	.067
Coblation	.358	.804	.270	.529
Electrocautery	.421	.465	.816	.348

Abbreviations: PM_2.5_, particulate matter <2.5 µm; SMPS-APS, scanning mobility particle sizer and an aerosol particle sizer.

**Table 2.** Aerosol Measurements for Each Procedure. ^a^

		Grimm				SMPS-APS			
Test	DustTrak PM_2.5_, µg/m^3^	PM_2.5_, µg/m^3^	Diameter, nm	Mass, µg/m^3^	Count per cm^3^	Diameter, nm	Geometric diameter, nm	Mass, µg/m^3^	Count per cm^3^
Blank	0.015 ± 0.096	0.538 ± 0.658	409 ± 970	7.27 ± 10.6	3.36 ± 1.74	208 ± 40	90.5 ± 2.46	0.701 ± 0.354	63.6 ± 51.8
Drilling	2.18 ± 7.71	5.68 ± 7.99	482 ± 127	130 ± 90.3	28.3 ± 46.9	227 ± 50.8	96.1 ± 1.49	1.04 ± 0.591	80.1 ± 35.1
Microdebrider	0.003 ± 0.021	0.345 ± 0.109	368 ± 41.6	0.900 ± 0.200	2.38 ± 1.10	149 ± 42.3	69.0 ± 1.37	0.461 ± 0.330	61.0 ± 20.3
Coblation	581 ± 1601	150 ± 263	398 ± 133	621 ± 1010	942 ± 1235	120 ± 14.1	108 ± 0.840	21.5 ± 13.6	23,164 ± 13,556
Electrocautery	1664 ± 2858	124 ± 89.4	337 ± 23.3	227.8 ± 201	1,726 ± 1,177	113 ± 10.5	103 ± 0.774	74.2 ± 38.0	76,468 ± 32,998
Electrocautery with suction	1409 ± 2764	114 ± 68.2	327 ± 22.4	134.6 ± 39.9	1,919 ± 1,342	128 ± 10.6	116 ± 0.786	67.3 ± 41.1	55,977 ± 32,713

Abbreviations: PM_2.5_, particulate matter <2.5 µm; SMPS-APS, scanning mobility particle sizer and an aerosol particle sizer.

a Values are presented as mean ± SD.

**Table 3.** Tukey-Adjusted *P* Values for Comparing the Measurements of Each Procedure With Blank (Background Condition).

Test	DustTrak PM_2.5_	Grimm PM_2.5_	Grimm total counts	SMPS-APS total counts
Drilling	.900	.870	.880	.890
Microdebrider	.900	.920	.900	.930
Coblation	.016	.004	.030	.040
Electrocautery	.001	.032	.001	.001
Electrocautery with suction	.005	.039	.001	.001

Abbreviations: PM_2.5_, particulate matter <2.5 µm; SMPS-APS, scanning mobility particle sizer and an aerosol particle sizer.

**Figure 4.**
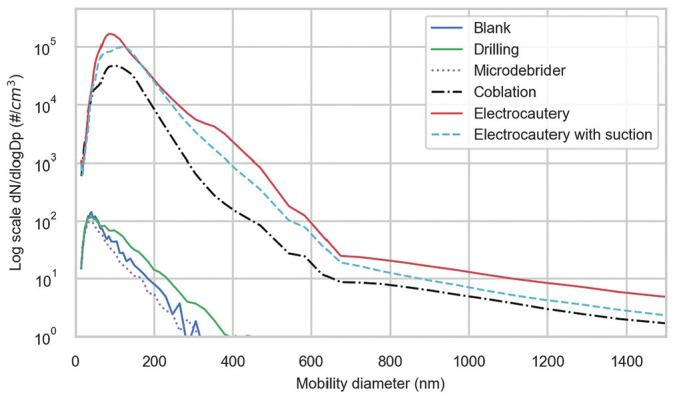
SMPS-APS particle size distribution for each procedure. The x-axis was limited for better representation of data. Note that the y-axis is log scale. SMPS-APS, scanning mobility particle sizer.

**Figure 5.**
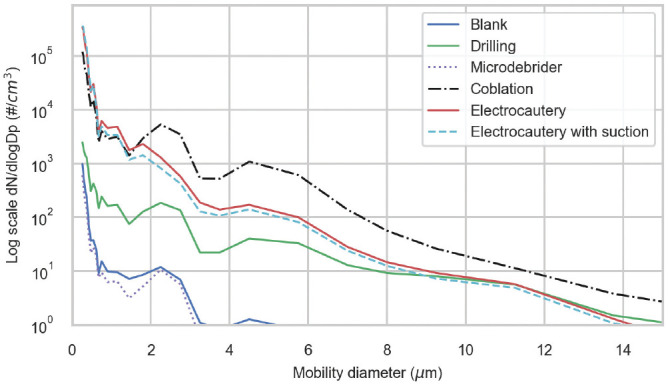
Grimm particle size distribution for each procedure. The x-axis was limited for better representation of data. Note that the y-axis is log scale.

**Figures 4** and **5** show the particle size distribution of the aerosols generated from each test. The results demonstrate that when compared with background, with a mean ± SD diameter of 208 ± 40 nm (**Table 2**), the heat-generated particles from coblation and electrocautery procedures yielded smaller particle sizes, with mean diameters of 120 ± 14.1 nm and 103 ± 10.5 nm (Tukey-adjusted *P* values <.023), respectively. Drilling and microdebrider, with mean diameters of 227 ± 50.8 nm and 149 ± 42.3 nm, did not show statistically significant differences as compared with background (Tukey-adjusted *P* values >.432 ). Since surgical masks are manufactured to filter particles >5 µm, we then calculated the relative percentage of particles <5 µm by procedure. Based on the SMPS-APS, >99.99% particles were <5 µm for all procedures. We also calculated the relative percentage of particles <0.7 µm, which is the limit of protection for N95 masks. More than 99.99% particles were also <0.7 µm for all procedures.

**Figure 6.**
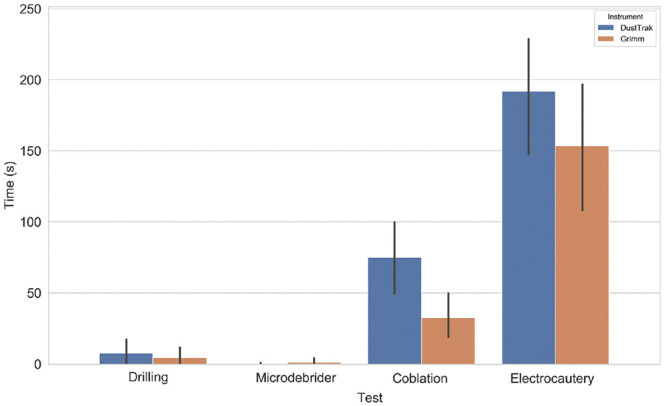
The mean decay time after each surgery. The error bars denote 1 SD.

**Figure 6** shows the time required after each procedure to reach background aerosol concentrations (decay time). The electrocautery procedure required the longest time, approximately 3 minutes, to reach background aerosol concentrations.

### Detection of Viral DNA and Infectious Virus

**Figure 7.**
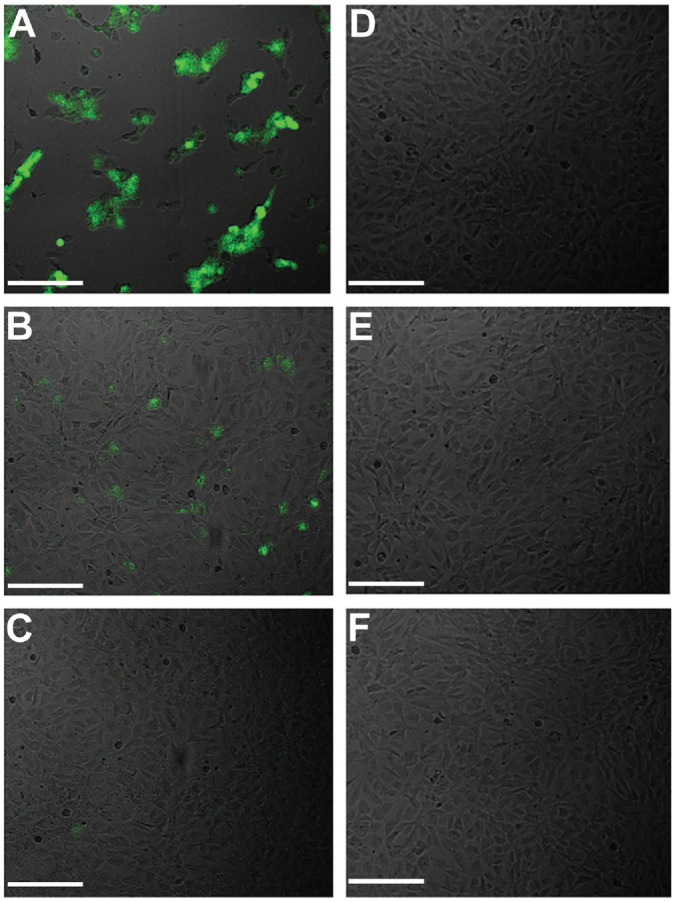
Assessment of viral infectivity by GFP fluorescence in tissue culture. NIH-3T3 cells were treated with (A-C) positive control samples or (D-F) condensate samples and incubated for 24 hours. Positive control inoculums were (A) 63,000 pfu, (B) 6300 pfu, and (C) 630 pfu of CMV-GFP. Representative condensate samples were (D) 52920 CTRL, (E) 60120 2A, and (F) 52920 1A. Images are merged GFP fluorescence and brightfield imaging. Scale bars indicate 100 µm. CMV, cytomegalovirus; GFP, green fluorescent protein; pfu, plaque-forming units.

Viral DNA was detected in 3 of the 12 condensates collected during coblation and 3 of the 16 condensates during drilling of tissue from CMV-infected mice (Supplementary Table S1). Viral DNA was not detected in condensate samples collected from noninfected mouse tissue for either technique. No viral DNA was detected in condensates collected during microdebrider or electrocautery use. The fact that viral DNA was not detected for microdebrider samples that had been injected with 6.3 × 10^4^ pfu of CMV immediately prior to the procedure suggests that the technique itself might be responsible for degrading the viral DNA. Infectious particles were not detected by GFP expression in tissue culture (**Figure 7**) or by GFP expression or CMV qPCR of culture cells after 3 days following any procedure. Tissue culture experiments resulted in confluent cell growth for all condensate-treated wells (**Figure 7E** and **7F**), whereas positive control wells treated with CMV-GFP virus showed lower cell density (**Figure 7A** and **7B**), indicating cell loss due to CMV infection. Similarly, positive control wells treated with CMV-GFP resulted in amplification of viral DNA by qPCR at levels well above input levels. These data indicate that infectious particles would have been detected in condensate-treated wells, if present at levels similar to or above the lowest levels of CMV included in positive controls.

## Discussion

The risk for patient transmission during an otolaryngology procedure will depend on the probability that sufficient quantities of viable virus will infect the health care worker. The results from our study demonstrate that a number of these procedures can generate relatively large concentrations of aerosolized particles and that a significant percentage are small enough to pass unimpeded through conventional surgical and even N95 masks. The highest concentration of aerosolized particles was found from electrocautery and coblation. The 1202-fold increase in aerosolized concentrations from electrocautery over background is consistent with the Carr et al study that measured the particle number concentrations of children who underwent electrocautery tonsillectomy.^20^ In an unpublished communication, an EDGE Electrosurgical Button Switch Pencil (Medtronics) and ENT Coblation Wand (Smith & Nephew) were tested on fresh bovine thymus and myocardial tissue to determine quantity of aerosolized particles.^21^ It reported a 240-fold reduction in particle concentration when the coblation wand was compared with the electrocautery device. We noted a much more modest reduction, which may be due to the lower-wattage setting that we used for our electrocautery experiments and the wider range of particle sizes measured with our instrumentation. The report cited a P-Trak Ultrafine Particle Counter with a range of 20 nm to 1 µm. Our SMPS-APS measures a wider distribution of aerosols, between 15 nm and 20 µm. Elmashae et al reported that electrocautery exhibited a peak in particle size between 60 and 150 nm, which agrees with our study’s peak for this procedure at 89.9 nm.^2^ We also found a modest reduction in aerosol concentrations when suction was applied in conjunction with electrocautery.

A surgical mask requires Food and Drug Administration clearance and protects the wearer from larger particles, >5 µm.^22^ A N95 mask is cleared by the National Institute for Occupational Safety and Health and Food and Drug Administration and will prevent aerosolized particles >0.7 µm.^23^ Our results indicate that 99.9% of particle counts for all the procedures are <0.7 µm. These results are consistent with others who reported that these particles can range from 10 nm to 1 µm.^2,24^ Mowbray et al searched the Cochrane Database, MEDLINE, PubMed, Embase Classic, Embase, and metaRegister of Controlled Trials and found 5 of 20 studies showing that diathermy or laser can produce ultrafine particles that are respirable in size.^25^ A powered air-purifying respirator (PAPR) may be a preferred option when performing some of these procedures in patients who are COVID-19 positive. A PAPR filters out contaminants in the air and uses a battery-operated blower to provide the user clean air through a tight-fitting respirator, hood, or helmet.^26^ It has a higher assigned protection factor than N95 or other air-purifying respirators, by a factor ≥25. An assigned protection factor is the ratio of pollutants outside the device (environment) to those inside the device (inhaled component).^26^

This approach would be difficult to use for otologic surgery, and there may be challenges with verbal communication. Bischoff et al reported that despite passing fit-testing, 10% of N95 respirator users encountered breakthrough with exposure to influenza virus as compared with full protection provided by a PAPR.^27^ However, there have been no controlled clinical trials comparing the efficacy of PARS with other modalities for SARS-CoV-2 or even for earlier pandemics (eg, SARS-CoV-1, Ebola, or MERS). A systematic review of SARS-CoV-2 observational and simulation studies failed to demonstrate differences in health care worker infection in cohorts using PAPRs versus other appropriate respiratory protection.^26^ A clinical trial to address this question is urgently needed.

In contrast to electrocautery and coblation, we did not detect significantly elevated concentrations of aerosols from microdebrider procedures. These results are consistent with Workman and colleagues’ assessment of microdebrider or cold instrumentation.^10^ They performed these procedures on 2 fresh-frozen heads in a dedicated surgical laboratory. Our results with drilling did not show a significant difference as compared with background levels. Workman et al also considered only particles between 1 and 10 µm. Perhaps their background aerosol concentrations were lower than those in our experiments. In addition, a human cadaveric sphenoid rostrum would contain much more bone stock than what could be drilled from the skull of an 8-day-old mouse, and this may have resulted in more mechanically generated particles in the 1- to 10-µm range.

An important component in viral transmissibility from these procedures is its viability as an aerosol. We were able to detect viral DNA in 3 of the 15 condensates for coblation. One might have expected to detect viral DNA from the electrocautery procedures given our reported high concentrations. Unlike coblation, which produces a plasma field at temperatures between 60° C and 70° C, electrocautery generates temperatures as high as 400° C to 600° C.^28^ This much higher temperature can denature any viral DNA. Johnson and Robinson measured aerosols from electrocautery and cooler drilling administered to known HIV-1–inoculated blood.^29^ Infectious HIV-1 was detected in the aerosolized collected viral culture media from drilling but not from electrocautery. Sawchuk et al reported aerosolized DNA following removal of plantar warts with electrocautery.^30^ These samples were obtained at a relatively low 6 W and with a sampling only 2 cm from the surgical site.

Despite the reassuring low evidence of viral transmission from electrocautery, the elevated concentrations and composition of the surgical plumes are concerning. These plumes may contain as much as 3 to 51 ppm of hydrogen cyanide, a known cardiotoxic compound, and 0.15 to 0.69 ppm of 1,3-butadiene, a known carcinogen.^31^ This surgical smoke can be toxic to patients as well as health care workers. Dobrogowski et al measured benzene and toluene in the urine of patients who underwent laparoscopic cholecystectomy and detected significantly higher concentrations after surgery than before, presumably from the absorption of surgical smoke.^32^ In the context of cigarette smoking, Tomita et al noted that electrocautery removal of 1 g of tissue has the mutagenic potential of smoking 3 to 6 cigarettes.^33^ Thus, surgeons, operating room staff, and patients should be aware of the harmful effects from surgical plume. Lower wattage and smoke evacuation systems should be used whenever possible.^20^

Somewhat surprising was our detection of viral DNA during the drilling procedures. This finding suggests the importance of an aerosol temperature that is not too high to denature the virus. Several studies have reported measurable aerosols following drilling procedures.^34,35^ Our results are particularly relevant given a recent publication confirming the presence of SARS-CoV-2 RNA detected in the middle ear and mastoid in 2 of 3 patients with COVID-19.^36^ Our findings would support the recommendation to consider COVID-19 preoperative screening and the use of appropriate precautions for potential aerosol and droplet generation for any middle ear or mastoid procedure.^36^ Other options to consider include modified drapes to reduce aerosol exposure and betadine irrigation during drilling to inactivate virus. Chari et al reported that a second suction and a barrier drape reduced the average measured aerosol to baseline levels.^37^ On the basis of an in vitro study demonstrating inactivation of SARS-CoV-2 and a small case series showing a significant reduction in COVID-19 viral loads in 2 of 4 patients using a povidone-iodine mouthwash, we have started to use 0.5% povidone-iodine irrigation during our mastoidectomy procedures.^38,39^

Samplers that are commonly used for bioaerosol sampling are not designed to collect nanosized viral aerosols.^14^ Hogan et al collected bacteriophages with 3 bioaerosol samplers—the AGI-30, the SKC BioSampler, and a frit bubbler—and noted that the collection efficiency for each system was <10% for particles in the range of 20 to 100 nm.^40^ We used a laminar-flow, water-based condensational particle growth tube collector. This device condenses water vapor onto a viral particle, creating droplets 2 to 5 µm in diameter. Using this same method, Pan et al demonstrated much greater collection of virus particles and efficiency of collecting viable virus—specifically, 10 to 100 times better than standard BioSamplers.^14^ Despite the greater sensitivity with this growth tube collector, we were not able to detect any viable virus.

We believe that this lack of viable virus reflects the very small quantities of aerosolized virus generated from these procedures. This observation is confirmed by the relatively low copy numbers in the detectable viral DNA and may mean that these procedures are low risk for transmitting infection. A limitation of our study, however, is that the collection time for each procedure was only 6 minutes 45 seconds. This duration was based on the limited tissue available for surgery from an 8-day-old mouse. A longer procedure may have enabled detection of more viral DNA and viable virus. We used a neonatal mouse infected with CMV because of our familiarity with this model and a mCMV virus, since it is not pathogenic to humans (biosafety 1 designation).^12,16,41^ The mCMV strain expresses a GFP during transcription that is readily noticeable during active infection. Tom and Mina suggested that patients with COVID-19 whose symptoms have resolved and who have a crossing threshold >34 are likely not to have meaningful or transmissible disease.^42^ Our studies indicate that these higher crossing thresholds were found in all our procedures. Future studies—perhaps with a more clinically relevant model, as in a hamster or mouse infected with SARS-CoV-2—may provide insight into the transmissible potential from aerosolizing procedures to health care workers.

## Conclusion

Coblation and electrocautery procedures generate >100-fold increases in aerosol concentrations over background; yet, only coblation and drilling produce aerosolized DNA samples. The absence of any viable infectious particles from all procedures is reassuring and may indicate a low potential for viral transmission. The high concentration of aerosols from coblation and electrocautery suggests the need for appropriate safeguards against particle exposure to health care workers. The presence of viral DNA from drilling and coblation procedures warrants the need for appropriate protection against droplet and aerosol exposure. Additional studies with this model in a SARS-CoV-2 animal model may provide insight into the relative risk from these procedures in patients infected with COVID-19.

## Supplemental Material

Supplemental_Figure_1 – Supplemental material for Airborne Aerosolized Mouse Cytomegalovirus From Common Otolaryngology Procedures: Implications for COVID-19 Infection Click here for additional data file. Supplemental material, Supplemental_Figure_1 for Airborne Aerosolized Mouse Cytomegalovirus From Common Otolaryngology Procedures: Implications for COVID-19 Infection by Tofigh Sayahi, Christopher Nielson, Yuan Yu, Kaden Neuberger, Michael Seipp, Matthew A. Firpo, Kerry Kelly and Albert H. Park in Otolaryngology–Head and Neck Surgery

Supplemental_Figure_2 – Supplemental material for Airborne Aerosolized Mouse Cytomegalovirus From Common Otolaryngology Procedures: Implications for COVID-19 Infection Click here for additional data file. Supplemental material, Supplemental_Figure_2 for Airborne Aerosolized Mouse Cytomegalovirus From Common Otolaryngology Procedures: Implications for COVID-19 Infection by Tofigh Sayahi, Christopher Nielson, Yuan Yu, Kaden Neuberger, Michael Seipp, Matthew A. Firpo, Kerry Kelly and Albert H. Park in Otolaryngology–Head and Neck Surgery

Supplementary_Table_1_8.1.20 – Supplemental material for Airborne Aerosolized Mouse Cytomegalovirus From Common Otolaryngology Procedures: Implications for COVID-19 Infection Click here for additional data file. Supplemental material, Supplementary_Table_1_8.1.20 for Airborne Aerosolized Mouse Cytomegalovirus From Common Otolaryngology Procedures: Implications for COVID-19 Infection by Tofigh Sayahi, Christopher Nielson, Yuan Yu, Kaden Neuberger, Michael Seipp, Matthew A. Firpo, Kerry Kelly and Albert H. Park in Otolaryngology–Head and Neck Surgery
