# Supplementary figures and images for: Airborne Aerosolized Mouse Cytomegalovirus From Common Otolaryngology Procedures: Implications for COVID-19 Infection
Source: Otolaryngol Head Neck Surg. 2020 Sep 15;164(3):547–55. doi: 10.1177/0194599820957966 (PMC7492827; doi:10.1177/0194599820957966)

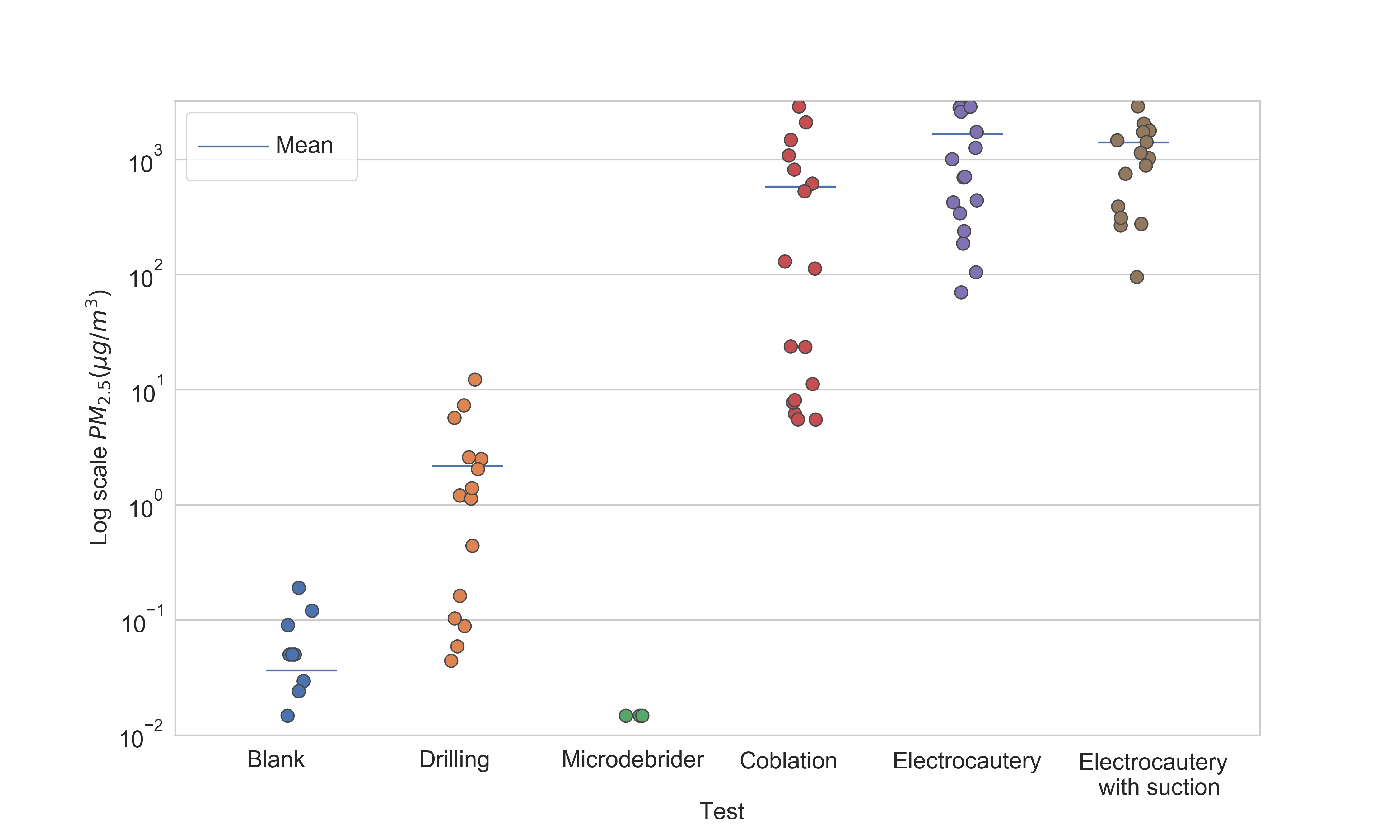

Supplement: Supplemental_Figure_1 – Supplemental material for Airborne Aerosolized Mouse Cytomegalovirus From Common Otolaryngology Procedures: Implications for COVID-19 Infection [file Supplemental_Figure_1.TIFF]

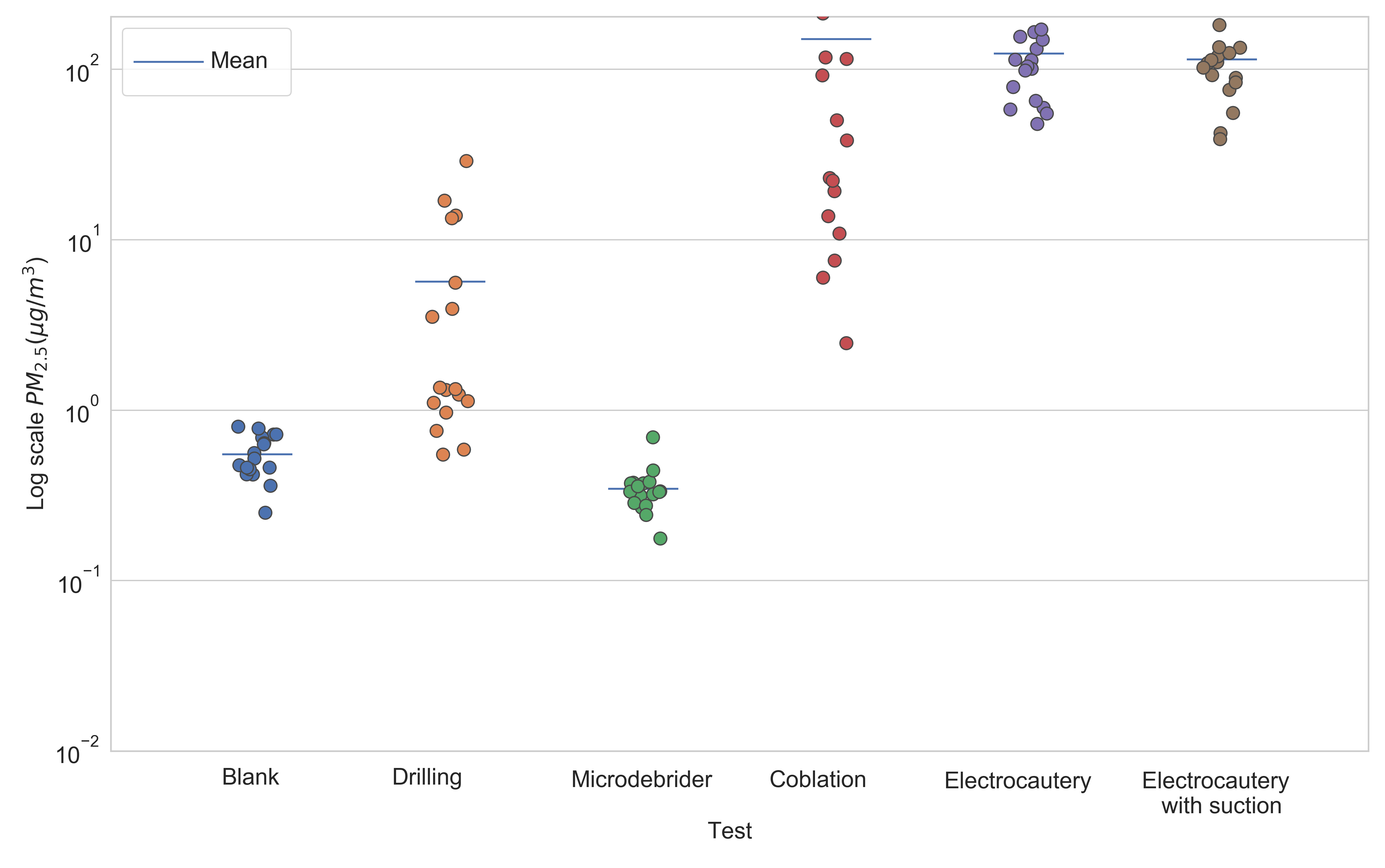

Supplement: Supplemental_Figure_2 – Supplemental material for Airborne Aerosolized Mouse Cytomegalovirus From Common Otolaryngology Procedures: Implications for COVID-19 Infection [file Supplemental_Figure_2.TIFF]
