# Supplementary material for: Airborne Aerosolized Mouse Cytomegalovirus From Common Otolaryngology Procedures: Implications for COVID-19 Infection
Source: Otolaryngol Head Neck Surg. 2020 Sep 15;164(3):547–55. doi: 10.1177/0194599820957966 (PMC7492827; doi:10.1177/0194599820957966)
Supplement: Supplementary_Table_1_8.1.20 – Supplemental material for Airborne Aerosolized Mouse Cytomegalovirus From Common Otolaryngology Procedures: Implications for COVID-19 Infection [file Supplementary_Table_1_8.1.20.docx]

Supplementary Table 1: Results of aerosol and tissue culture qPCR analysis

|  |  |  | ge/Rx^a^ | | |
| --- | --- | --- | --- | --- | --- |
| Procedure | Treatment | Sample Name | condensate | 3-day tissue culture cells | 3-day tissue culture cells+supernatant |
| Cautery | IC saline | 52920 1D | ND | ND | ND |
| Cautery | IC saline | 52920 2D | ND | ND | ND |
| Cautery | IC saline | 52920 3D | ND | ND | ND |
| Cautery | IC CMV | 60120 1D | ND | ND | ND |
| Cautery | IC CMV | 60120 2D | ND | ND | ND |
| Cautery | IC CMV | 60120 3D | ND | ND | ND |
| Cautery | IC CMV | 60120 4D | ND | ND | ND |
| Cautery | IC CMV | 60120 5D | ND | ND | ND |
| Cautery | IC CMV | 60120 6D | ND | ND | ND |
| Cautery | IC CMV | 60420 1D | ND | ND | ND |
| Cautery | IC CMV | 60420 2D | ND | ND | ND |
| Cautery | IC CMV | 60420 3D | ND | ND | ND |
| Cautery | IC CMV | 60420 4D | ND | ND | ND |
| Cautery | IC CMV | 60420 7D | ND | ND | ND |
| Cautery | IC CMV | 60420 8D | ND | ND | ND |
| Cautery | IC CMV | 60420 9D | ND | ND | ND |
| Coblation | IC saline | 52920 1C | ND | ND | ND |
| Coblation | IC saline | 52920 2C | ND | ND | ND |
| Coblation | IC saline | 52920 3C | ND | ND | ND |
| Coblation | IC CMV | 60120 1C | ND | ND | ND |
| Coblation | IC CMV | 60120 2C | ND | ND | ND |
| Coblation | IC CMV | 60120 3C | ND | ND | ND |
| Coblation | IC CMV | 60120 5C | ND | ND | ND |
| Coblation | IC CMV | 60120 6C | ND | ND | ND |
| Coblation | IC CMV | 60420 1C | ND | ND | ND |
| Coblation | IC CMV | 60420 2C | ND | ND | ND |
| Coblation | IC CMV | 60420 3C | ND | ND | ND |
| Coblation | IC CMV | 60420 4C | ND | ND | ND |
| Coblation | IC CMV | 60420 7C | 48 | ND | ND |
| Coblation | IC CMV | 60420 8C | 193 | ND | ND |
| Coblation | IC CMV | 60420 9C | 63 | ND | ND |
| Drill | IC saline | 52920 1A | ND | ND | ND |
| Drill | IC saline | 52920 3A | ND | ND | ND |
| Drill | IC CMV | 60120 1A | ND | ND | ND |
| Drill | IC CMV | 60120 2A | ND | ND | ND |
| Drill | IC CMV | 60120 3A | ND | ND | ND |
| Drill | IC CMV | 60120 4A | ND | ND | ND |
| Drill | IC CMV | 60120 5A | ND | ND | ND |
| Drill | IC CMV | 60120 6A | ND | ND | ND |
| Drill | IC CMV | 60420 10A | ND | ND | ND |
| Drill | IC CMV | 60420 1A | ND | ND | ND |
| Drill | IC CMV | 60420 2A | 606 | ND | ND |
| Drill | IC CMV | 60420 3A | ND | ND | ND |
| Drill | IC CMV | 60420 4A | 201 | ND | ND |
| Drill | IC CMV | 60420 5A | 144 | ND | ND |
| Drill | IC CMV | 60420 6A | ND | ND | ND |
| Drill | IC CMV | 60420 7A | ND | ND | ND |
| Drill | IC CMV | 60420 8A | ND | ND | ND |
| Drill | IC CMV | 60420 9A | ND | ND | ND |
| Microdebrider | IC saline | 52920 1B | ND | ND | ND |
| Microdebrider | IC saline | 52920 2B | ND | ND | ND |
| Microdebrider | IC saline | 52920 3B | ND | ND | ND |
| Microdebrider | IC CMV | 60120 1B | ND | ND | ND |
| Microdebrider | IC CMV | 60120 2B | ND | ND | ND |
| Microdebrider | IC CMV | 60120 3B | ND | ND | ND |
| Microdebrider | IC CMV | 60120 4B | ND | ND | ND |
| Microdebrider | IC CMV | 60120 5B | ND | ND | ND |
| Microdebrider | IC CMV | 60120 6B | ND | ND | ND |
| Microdebrider | IC CMV | 60420 1B | ND | ND | ND |
| Microdebrider | IC CMV | 60420 2B | ND | ND | ND |
| Microdebrider | IC CMV | 60420 3B | ND | ND | ND |
| Microdebrider | IC CMV | 60420 4B | ND | ND | ND |
| Microdebrider | IC CMV | 60420 7B | ND | ND | ND |
| Microdebrider | IC CMV | 60420 8B | ND | ND | ND |
| Microdebrider | IC CMV | 60420 9B | ND | ND | ND |
| Microdebrider x 14 mins | injected spleen (630,000 pfu) | 60420 1 CTRL | ND | ND | ND |
| Microdebrider x 14 mins | injected spleen (630,000 pfu) | 60420 2 CTRL | ND | ND | ND |
| Microdebrider x 14 mins | IC CMV | 60420 10B | ND | ND | ND |
| Microdebrider x 14 mins | IC CMV | 60420 6B | ND | ND | ND |
| Microdebrider/Coblation/Cautery | IC CMV | 60420 5D | ND | ND | ND |
| pre-procedure condensate |  | 52920 CTRL | ND | ND | ND |
| post-procedure condensate |  | 60120 CTRL | ND | ND | ND |
| Spleen | IC CMV | 60120 5 spleen | ND | ND | ND |
| Spleen | IC CMV | 60120 6 spleen | ND | ND | ND |
| Spleen | IC CMV | 60420 1 Spleen | ND | ND | ND |
| Spleen | IC CMV | 60420 10 Spleen | ND | ND | ND |
| Spleen | IC CMV | 60420 2 Spleen | 97 | ND | ND |
| Spleen | IC CMV | 60420 3 Spleen | ND | ND | ND |
| Spleen | IC CMV | 60420 4 Spleen | ND | ND | ND |
| Spleen | IC CMV | 60420 5 Spleen | ND | ND | ND |
| Spleen | IC CMV | 60420 6 Spleen | ND | ND | ND |
| Spleen | IC CMV | 60420 7 Spleen | ND | ND | ND |
| Spleen | IC CMV | 60420 8 Spleen | ND | ND | ND |
| Spleen | IC CMV | 60420 9 Spleen | ND | ND | ND |
| Positive Control | 6300 pfu CMV |  |  | 2769976 | 29176119 |
| Positive Control | 6300 pfu CMV |  |  | 3202138 | 32670335 |
| Positive Control | 630 pfu CMV |  |  | 339101 | 61178107 |
| Positive Control | 630 pfu CMV |  |  | 406865 | 53041786 |
| Positive Control | 63 pfu CMV |  |  | - | 53310793 |
| Positive Control | 63 pfu CMV |  |  | - | 34016669 |

^a^Murine CMV genome equivalents per reaction: signal in replicate samples, with mean signal above LOD; ND = not detected

IC: intracerebral

ND: Not detectable
